# Comparison of Different Drying Methods on the Volatile Components of Ginger (*Zingiber officinale* Roscoe) by HS-GC-MS Coupled with Fast GC E-Nose

**DOI:** 10.3390/foods11111611

**Published:** 2022-05-30

**Authors:** Dai-Xin Yu, Sheng Guo, Jie-Mei Wang, Hui Yan, Zhen-Yu Zhang, Jian Yang, Jin-Ao Duan

**Affiliations:** 1 National and Local Collaborative Engineering Center of Chinese Medicinal Resources Industrialization and Formulae Innovative Medicine, Jiangsu Collaborative Innovation Center of Chinese Medicinal Resources Industrialization, Nanjing University of Chinese Medicine, Nanjing 210023, China; yudaixin0616@163.com (D.-X.Y.); wjm991106@163.com (J.-M.W.); yanhui@njucm.edu.cn (H.Y.); 15251766992@163.com (Z.-Y.Z.); dja@njucm.edu.cn (J.-A.D.); 2 State Key Laboratory of Dao-di Herbs Breeding Base, National Resource Center for Chinese Materia Medica, China Academy of Chinese Medical Sciences, Beijing 100700, China; yangchem2012@163.com

**Keywords:** ginger, drying methods, HS-GC-MS, fast GC e-nose, volatile components, flavor profiles

## Abstract

Ginger (*Zingiber officinale* Roscoe) is one of the most popular spices in the world, with its unique odor. Due to its health benefits, ginger is also widely used as a dietary supplement and herbal medicine. In this study, the main flavor components of gingers processed by different drying methods including hot air drying, vacuum drying, sun-drying, and vacuum-freeze drying, were identified on the basis of headspace-gas chromatography coupled with mass spectrometry (HS-GC-MS) and fast gas chromatography electronic-nose (fast GC e-nose) techniques. The results showed that the ginger dried by hot air drying exhibited high contents of volatile compounds and retained the richest odor in comparison with those dried by other methods, which indicated that hot air drying is more suitable for the production of dried ginger. Sensory description by fast GC e-nose exhibited that ginger flavor was mainly concentrated in the spicy, sweet, minty, fruity, and herbaceous odor. The relative content of the zingiberene was significantly higher in the hot air drying sample than those by other methods, suggesting that dried ginger by hot air drying can retain more unique spicy and pungent odorants. Furthermore, the results of chemometrics analyses showed that the main variance components among the samples by different drying methods were *α*-naginatene, (+)-cyclosativene, and sulcatone in HS-GC-MS analysis, and *α*-terpinen-7-al, dimethyl sulfide, and citronellal in fast GC e-nose analysis. For comparison of fresh and dried gingers, terpinolene, terpinen-4-ol, 2,4-decadienal, (E, Z)-, and linalool were considered the main variance components. This study generated a better understanding of the flavor characteristics of gingers by different drying methods and could provide a guide for drying and processing of ginger.

## 1. Introduction

Ginger (*Zingiber officinale* Roscoe) is one of the hot spices belonging to the Zingiberaceae family [1]. Due to its high nutritional and medicinal values, ginger is also extensively used as a dietary supplement, herbal medicine, and flavoring agent [2]. In addition to being eaten fresh, ginger is often processed into dried products and added to many desserts and beverages, such as ginger tea, ginger candies, and ginger beer [3,4]. Modern studies found that gingerols, shogaols, terpenes, and sugars are the key bioactive constituents of ginger, and moreover, these compounds are considered as the essential part of the pungency and aromatic flavor in ginger [5,6,7]. Some biological activities of ginger, including antioxidant [8], anti-inflammatory [9], antimicrobial activity [10], and immune-modulatory activity [11], have been reported, which have indicated the benefits of ginger and its products for humans.

To date, many studies have revealed that different drying methods can make different effects on the aroma compositions of food or herbs. During the drying process, certain losses and conversions of volatile compounds can occur, which affects the quality and sensory properties of the samples [12]. Volatile organic compounds (VOCs), which mainly consist of monoterpenes and sesquiterpenes, are considered important factors in determining the quality and fragrant behavior of dried ginger (DG) [13]. Generally, DG with abundant aroma is also more popular among consumers and producers. Several studies have evaluated the changes of volatile components of ginger by different drying methods [14,15]. However, these studies were usually performed using gas chromatography coupled with mass spectrometry (GC-MS), which could not avoid the tedious sample preparation and time-consuming analytical processes. In addition, conventional GC-MS analysis does not provide exact sensory information about the odor [16]. Therefore, the development of a rapid aroma preparation method and flavor sensory description is very important for the identification of the overall odor information of gingers by different drying methods.

As a rapid and green extraction method, the headspace technique (HS) has been widely combined with GC-MS [17]. Compared to traditional extraction methods for volatile oils, the HS technique could mainly acquire sufficient odor composition and reduce the analysis time and pollution from organic reagents [18]. HS-GC-MS can realize the separation, identification, and quantification of aromatic compounds of DG through GC system and ion fragmentation. However, it does not possess the function to translate the volatile components into sensory perceptions and provide flavor description. With the development of electronic and sensory technology, electronic nose (e-nose) has gradually replaced the traditional judgment and been applied for the sensory evaluation of food. In recent years, the combination of headspace, e-nose, and gas chromatography, called fast GC e-nose, has been successfully developed to characterize the composition of flavor components in food or herbs with adequate odor [19]. Due to the capability of HS phase and GC system, fast GC e-nose can identify unknown odor types in a very short time (1–3 min) and provide qualitative analysis of volatile components [16,20]. As an accurate, nondestructive, rapid, and objective detector, the fast GC e-nose has been used to assess the aroma profiles of Chinese jujubes by various drying programs [21] and explore the aroma dynamic characteristics during the drying process of green tea [22]. In addition, this flavor instrument is also used in geographical origin traceability [23,24]. Although previous studies have revealed the characteristics of the volatile components of ginger [25,26], the flavor description of ginger by different drying methods has not been carried out.

In this study, to characterize the effects of the drying methods on volatile components and flavor composition of ginger, two odor analysis strategies, HS-GC-MS and fast GC e-nose, were adopted. The processing methods of ginger included hot air drying (HAD, with the temperatures at 50, 60 and 70 °C, respectively), vacuum drying (VD, with the temperatures at 50, 60 and 70 °C, respectively), sun-drying (SD), and vacuum-freeze drying (VFD). Fresh ginger (FG) was used to compare the flavor changes before and after the drying process. Multivariate statistical analyses including principal component analysis (PCA) and partial least-squares discriminant analysis (PLS-DA) were used to investigate the potential differential flavor components of different dried gingers. The results are expected to provide further insight in flavor compounds and sensory evaluation in gingers processed by different drying methods.

## 2. Materials and Methods

### 2.1. Ginger Samples

FG samples were collected in October 2021 from the main ginger production region (Luoping County, Yunnan Province) in China, and these samples were identified as the fresh rhizomes of *Zingiber officinale* Roscoe by Prof. Jin-ao Duan from Nanjing University of Chinese Medicine. All fresh ginger rhizomes were washed, and the mud, sand, and fibrous roots on the surface were removed. After cleaning, these samples were cut into 3–5 mm thick slices for subsequent drying studies.

### 2.2. Drying Process

#### 2.2.1. Hot Air Drying Process (HAD)

The FG slices were placed on a clean tray and directly dried in an electric thermostatic drying oven (DHA-9070A, Shanghai Jinghong Experimental Equipment Co., Ltd., Shanghai, China) at constant temperatures of 50, 60 and 70 °C. All samples were dried to a moisture content of approximately 10%.

#### 2.2.2. Vacuum Drying Process (VD)

The vacuum drying process was carried out in a vacuum drying oven (DZF-6090, Shanghai Augem Instrument Manufacturing Co., Ltd., Shanghai, China). The FG slices were placed on a clean tray and dried with vacuum pressure set of 0.1 MPa at 50, 60 and 70 °C. All samples were dried to a moisture content of approximately 10%.

#### 2.2.3. Sun Drying (SD)

The FG samples were evenly laid flat on the trays and left to dry in a sunny and well-ventilated location. The average temperature was 25 °C, the drying time lasted for 3 days, and the moisture content was controlled at approximately 14%.

#### 2.2.4. Vacuum-Freeze Drying (VFD)

The FG slices were firstly pre-frozen in an ultra-low temperature refrigerator at −80 °C for 12 h, then quickly placed into a freeze-dryer (Labconco, FreeZone 6 freeze-dryer, Kansas City, MO, USA). The cold trap temperature and vacuum pressure were set to −80.0 °C and 1.0 Pa, respectively. The whole drying time lasted 48 h, and the moisture content was controlled at approximately 10%.

### 2.3. Preparation of Dried Ginger Powder

DG samples prepared by different drying methods were crushed into powder by a high-speed grinder (FW-80, Tianjin Taisite Instrument Co., Tianjin, China), and then these powders were all passed through 50 mesh sieves for experimental use.

### 2.4. Color Measurements

The color characteristics of the FG and DG samples obtained by different drying methods (HAD, VD, SD, and VFD) were measured by a chroma analyzer (CM-5, KONICA MINOLTA, Tokyo, Japan). The spectrophotometer was first calibrated by a white plate, and the powder was evenly flat in the measuring dish, keeping the same weight and thickness of all samples. Six determinations were carried out for each drying method of ginger samples. According to the CIELAB color space theory of the International Commission on Illumination, the parameters of L* value (darkness to brightness), a* value (greenness to redness), and b* value (blueness to yellowness) were collected [27]. The total color difference (∆E) was used to describe the color change of DG with the following equation [12]:

∆E = [(L* − *L*_0_*)^2^ + (a* − *a*_0_*)^2^ + (b* − *b*_0_*)^2^]^1/2^ (1)

The values of *L*_0_*, *a*_0_*, and *b*_0_* were measured by fresh ginger.

The values of chroma and hue angle (H°) were used to describe the abundance of color and were calculated as follows:

Chroma = (a*^2^ + b*^2^)^1/2^ (2)

H° = tan^−1^(b*/a*) when a* ≥ 0 and b* ≥ 0 (3)

H° = 180 + tan^−1^(b*/a*) when a* < 0 (4)

### 2.5. Analysis of Volatile Components by HS-GC-MS

The HS-GC-MS analysis was carried out in an Agilent 7890B gas chromatograph (Agilent Technologies, Santa Clara, CA, USA) coupled to an Agilent 7000C triple quadrupole mass spectrometer (Agilent Technologies, Santa Clara, CA, USA), which was equipped with an electron impact (EI) ionization chamber and worked in full scan mode. A HP-5MS quartz capillary column (30 m × 0.25 mm, 0.25 µm) was used for separation. Headspace extraction was performed by an Agilent 7697A autosampler (Agilent Technologies, Santa Clara, CA, USA).

HS conditions were as follows: 0.3 g of ginger powder was accurately weighed and added into 20 mL headspace vials. Then, the vials were capped by a special PTFE-silicon spacer and incubated at 70 °C for 10 min, awaiting injection. In addition, the quantitative tube temperature and the transfer tube temperature were set to 85 and 100 °C, respectively. The injection time was maintained at 0.5 min.

For GC conditions, the temperature of the injection port was set to 250 °C, the split ratio was set to 50:1, and the carrier gas was helium (99.999% pure) with a flow rate of 1.0 mL/min. The oven temperature program started from 50 °C, was held for 1 min, and increased to 82 °C at a rate of 8 °C/min, then increased to 106 °C at a rate of 3 °C/min, was subsequently heated to 145 °C at a rate of 4 °C/min, and was finally ramped at a rate of 15 °C/min to 230 °C, remaining there for 5 min. The total run time of the GC program was 33 min. For MS conditions, the electron ionization (EI) energy was 70 eV, and the ion source temperature was 230 °C. The transfer line temperature was set at 280 °C. A full scan mode was operated at a mass range of 50.0–500.0 amu with a quadrupole temperature of 150 °C.

Volatile components were processed in Agilent MassHunter Qualitative Analysis (B.07.00) software. R match scores and retention index (RI) values in the NIST Mass Spectral Library (NIST 14.0, National Institute of Standards and Technology, Gaithersburg, MD, USA) were used to identify unknown compounds. The RI value of each component was calculated using an n-alkane solution (C_6_ to C_16_) under the same conditions. By comparing the measured and published literature RI values and combining them with the highest R-match scores, the compound information was finally determined. Generally, the standard for determination of volatile compounds is that the difference between the experimental RIs and the theoretical values does not exceed 30 [28]. For quantitative analysis of the different components, 15 µL of n-decyl hydride (0.4 mg/mL) was added to each sample as an internal standard, and the relative content of each component was determined by the ratio of the peak area measured to that of the internal standard.

### 2.6. Analysis of Flavor Compounds by Fast GC E-Nose

#### 2.6.1. Sample Incubation

A headspace autosampler (PAL-RSI, Alpha M.O.S., Toulouse, France) was used to extract the flavor compounds of ginger samples. Briefly, 0.1 g DG powders were added to 20 mL specialized vials for headspace extraction. To allow the odor to saturate the headspace bottles, the incubation temperature was set to 55 °C for 10 min, with a stirring speed of 500 rpm.

#### 2.6.2. Acquisition of Flavor Components

The gas incubated by headspace was injected directly into the fast GC e-nose system for odor analysis. The odor information was collected by a Heracles NEO e-nose system (Alpha M.O.S., Toulouse, France), which was combined with fast gas chromatography and the sensory functions of electronic nose. Two different polarity columns (MXT-5: a nonpolar column, 10 m × 0.18 mm × 0.4 µm; MXT-1701: a low-polar column, 10 m × 0.18 mm × 0.4 µm) were simultaneously used to monitor the concentrated odor and realize rapid separation. The flavor compounds were detected by two flame ionization detectors (FIDs).

The injection volume was set to 1000 µL at a speed of 125 µL/s, and the inlet temperature and pressure were set as 200 °C and 10 kPa, respectively. The following oven temperature program was used: initial temperature of 50 °C (2 s), increased to 100 °C with 6 °C/s, then heated to 200 °C with 2 °C/s, and finally ramped at 1 °C/s to 250 °C (10 s). Hydrogen was used as the carrier gas at a constant flow of 1.0 mL/min. Each sample was repeatedly measured three times, following the above conditions.

#### 2.6.3. Qualitative Analysis of Flavor Components

N-Alkanes C_6_ to C_16_ (Restek France, Lisses, France) were detected as the same method as ginger samples and then RI values of each unknown component were calculated according to MXT-5 and MXT-1701 columns. The flavor compounds were identified by comparing the measured RI values with Kovats relative retention index in the Arochem Base database.

### 2.7. Statistical Procedures

Principal component analysis (PCA), partial least squares discriminant analysis (PLS-DA), and orthogonal partial least squares discriminant analysis (OPLS-DA) were performed using SIMCA-P software (Version 14.1, Umetrics, Sweden). IBM SPSS Statistics 26.0 software (SPSS Inc., Chicago, IL, USA) was used for significant difference analysis by Waller–Duncan’s multiple-range text (*p* < 0.05 as the significance threshold). Histogram and Venn diagram were plotted in Origin 2021 pro (Microcal Software, Inc., Northampton, NC, USA).

## 3. Results and Discussion

### 3.1. Analysis of the Appearance Characteristics and Color Changes of Dried Gingers

**Figure 1:**
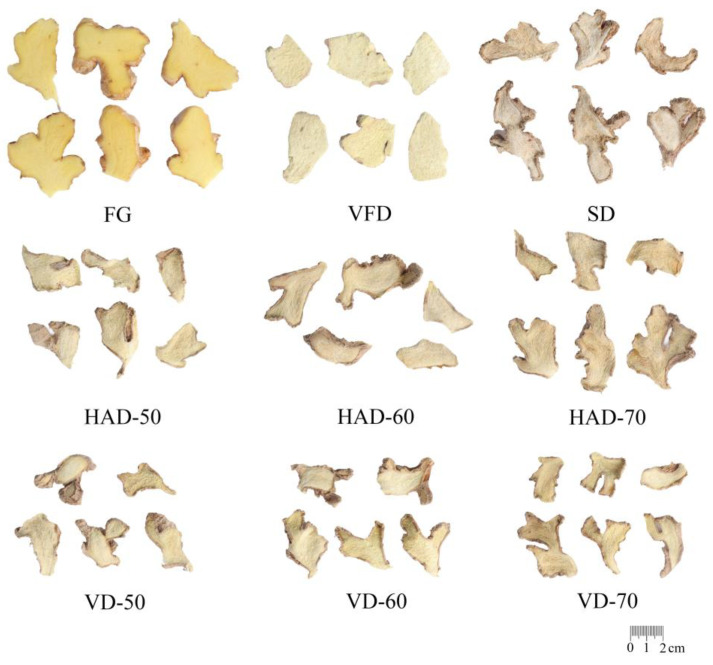
Appearance of ginger samples by different drying methods. HAD-50: hot air drying at 50 °C; HAD-60: hot air drying at 60 °C; HAD-70: hot air drying at 70 °C; VD-50: vacuum drying at 50 °C; VD-60: vacuum drying at 60 °C; VD-70: vacuum drying at 70 °C; VFD: vacuum freeze drying; SD: sun drying; FG: fresh ginger.

The appearance characteristics of FG and DG by different drying methods (HAD, VD, SD, and VFD) are shown in Figure 1. In terms of shape, DG by VFD kept almost the same shape as fresh ginger, while those by HAD, VD, and SD were all shrunken. The color of DG also changed obviously by different drying conditions through visual observation. Compared to FG, the DG treated by VFD was significantly lighter in color, and the sample obtained by SD was close to white, while the samples obtained by HAD and VD were similar in color (light yellowish). Furthermore, the color of DG was quantified and characterized on the basis of CIELAB chromaticity space theory [25]. The chroma data including L*, a*, b*, chroma, hue angle, and ∆E values are listed in Table 1. The L* values of the dried gingers obtained with different methods were increased to different degrees except for HAD-70, and VFD gave the highest L* value, which indicated the brightest color. Ginger by SD showed the lowest a* value compared to FG, indicating that its color was far from the original yellow of FG. The values of chroma and hue angle suggested that ginger by VFD was colorful abundance. The ∆E is an important indicator used to measure the color changes of DG before and after processing. The ∆E values of samples by VFD and SD samples were significantly higher than those of other processing methods, while ginger by HAD was closed to FG, indicating that vacuum freeze-drying and sun-drying had the greatest effect on color changes of DG, which is consistent with the conclusion reached by visual inspection.

### 3.2. HS-GC-MS Analysis

#### 3.2.1. Identification and Analysis of Volatile Components

**Figure 2:**
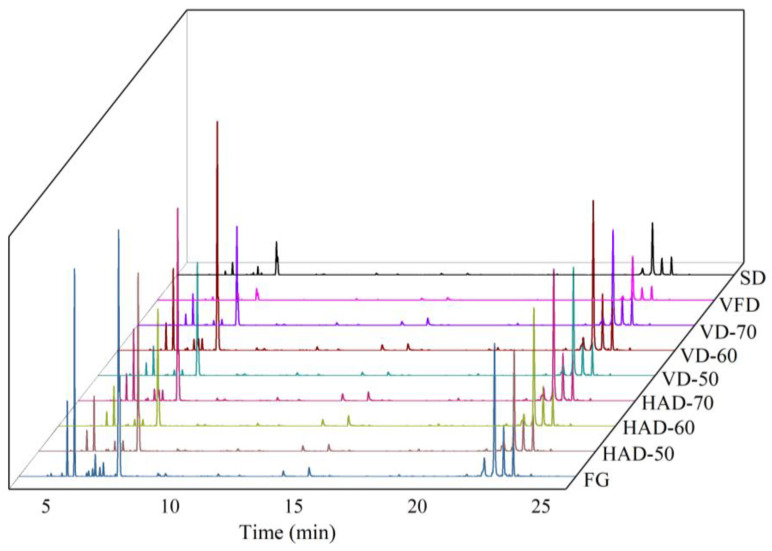
The total ion chromatography (TIC) plots of the samples obtained by different drying methods using HS-GC-MS.

**Figure 3:**
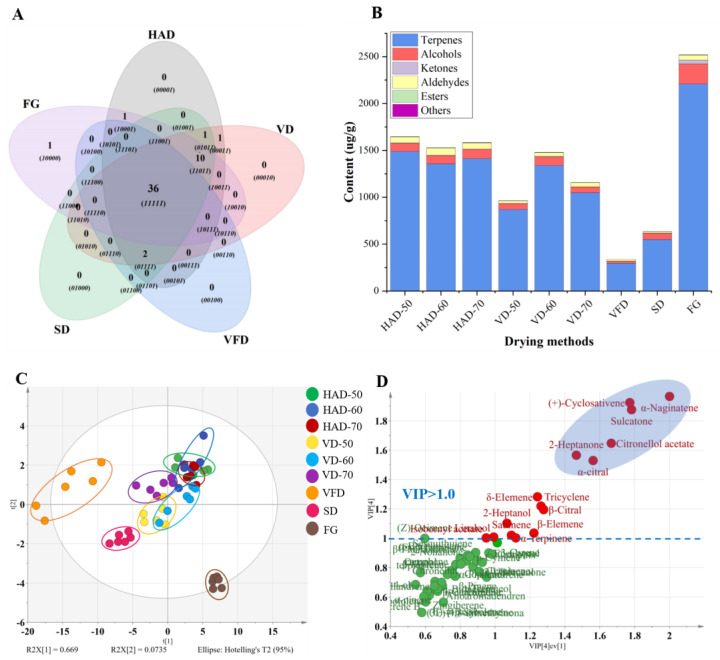
The Venn diagram of the volatile distribution for the fresh and dried gingers (**A**). The contents of different types of volatile compounds by different drying methods in ginger (**B**). Score plots of the PCA model (**C**) and VIP values of PLS-DA model (**D**) of different drying methods in gingers by HS-GC-MS.

In the present research, a total of 52 volatile components were identified from fresh and dried gingers. The total ion chromatography (TIC) plots and detailed information of various compounds are shown in Figure 2 and Table 2. Among these compounds, terpenes (37) were confirmed to be the main constituents of ginger flavor, accounting for more than 65% in all the volatiles, followed by ketones (4), aldehydes (4), alcohols (3), esters (2), and alkenes (2). As shown in the Venn plot (Figure 3A), a total of 36 common components were detected by different drying methods. The whole distribution of volatile components showed that the main compounds of FG and DG were zingiberene, *β*-phellandrene, *β*-bisabololene, *β*-sesquiphellandrene, *α*-curcumene, eucalyptol, camphene, *α*-pinene, and *α*-phellandrene, which covered the main aroma information of ginger. The relative contents of zingiberene and *β*-phellandrene were relatively high, both accounting for more than 25% in each tested compound, which is consistent with the result of An et al. [29]. Different drying methods showed various effects on the volatile components of ginger. For the hot-air-dried ginger, 51 aroma compounds were extracted and identified. Hot air drying at 50, 60 and 70 °C had little effect on the types of volatile composition, but there were some differences in their contents (Table S1). We found that the contents of monoterpenes (*β*-phellandrene, camphene, *α*-pinene) were significantly higher in the samples dried at 50 °C, while the contents of sesquiterpenes (zingiberene, *β*-bisabololene, *β*-sesquiphellandrene, α-curcumene) were more abundant in the samples dried at 60 °C. This suggested that the synthesis of short-chain olefins may be promoted with the increasing temperature. However, the contents of these monoterpenes slightly increased and sesquiterpenes slightly decreased when the temperature increased to 70 °C, which could be attributed to the degradation of sesquiterpenes to monoterpenes due to excessively high temperature [29].

In the samples dried by VD, SD, and VFD, there were 50, 49, 37 aroma compounds identified, respectively. As shown in Figure 3B, FG contained the highest contents of volatile compounds, followed by HAD, VD, and SD gingers, while VFD ginger presented the lowest volatile content. Compared to FG, the relative contents of volatile components were significantly decreased after heat treatment (hot air or vacuum drying), which could be attributed to the effects of the hot air and vacuum; this was also demonstrated by Ding et al. [26]. In addition, ginger dried by VFD showed significant decrease in categories and contents of volatile compositions to FG, which is similar to the results of An et al. [29]. We also found that the reduced volatile components were mainly concentrated in monoterpenes, and a total of eight monoterpenes, namely, tricyclene, *α*-thujene, sabinene, 3-carene, *α*-terpinene, (Z)-ocimene, *γ*-terpinene, and (-)-camphor, were not detected. Studies have shown that low-temperature conditions of freeze-drying may result in a decrease in enzymatic activity and downregulation or disruption of metabolic pathways, leading to a significant decline in volatile compounds [30]. It may be a reason for the decrease in total volatiles of dried ginger by VFD. Furthermore, volatile components of frozen samples are more likely to lose by sublimation in vacuum conditions, which may be another reason for the low contents of volatile compounds in freeze-dried gingers. In general, the conventional hot air-drying method was able to retain the maximum varieties and contents of volatile components of ginger, suggesting that hot air drying is more suitable for the production of dried ginger.

#### 3.2.2. Discrimination of the Dried Gingers by PCA and PLS-DA Analysis

To further explore the differences among the FG and DGs dried by HAD, VD, SD, and VFD, PCA and PLS-DA models were used for analysis. PCA and PLS-DA are both pattern recognition methods, and they can differentiate the samples on the basis of complex chemical information. In this study, the total values of the first two PCs were 66.9% and 7.3%, with R^2^X = 0.808 and Q^2^ (cum) = 0.688, indicating that the total variation could be better explained and predicted, respectively. As shown in the score plots (Figure 3C), the FG could be significantly distinguished from the processed samples, indicating that the volatile chemical profiles differed greatly between the fresh and dried gingers. For different drying methods, gingers by VFD and SD could be categorized separately, while the distinction between HAD and VD samples was not obvious. To determine the key volatile components affecting the differences among these samples, the PLS-DA model was employed. Permutation tests of 200 times showed that the intercepts of R^2^Y (0.165) and Q^2^Y (−0.201) were less than 0.3 and 0.05, indicating that this model was reliable and that no overfitting phenomenon existed. The variable importance factors (VIP) are distributed in Figure 3D, and 15 volatile components (VIP > 1) with the highest discrimination potential were found. Among them, *α*-naginatene, (+)-cyclosativene, sulcatone, citronellol acetate, 2-heptanone, and *α*-citral were considered to be the most critical factors that influenced the distinction of gingers by different drying methods.

### 3.3. Fast GC E-Nose Analysis

#### 3.3.1. Analysis of Flavor Components

**Figure 4:**
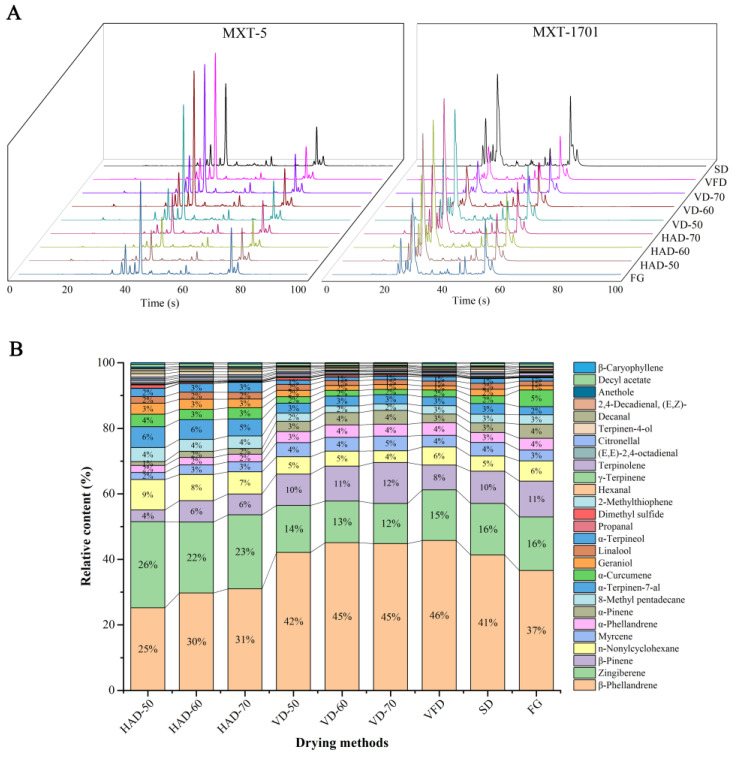
The chromatographic behaviors of flavor components in MXT-5 and MXT-1701 columns by fast GC e-nose (**A**). The histogram of odor abundance of different components in the gingers processed by various drying methods (**B**).

The flavor components of ginger by different drying methods were sniffed by fast GC e-nose. As a new type of rapid odor analyzer, the fast GC e-nose can imitate the human sense of smell to provide sensory information of volatile components. In this work, the flavor compounds were separated using two columns (MXT-5 and MXT-1701). According to the chromatographic behaviors (Figure 4A), the MXT-5 column was more effective in separation of ginger flavor compositions. Therefore, the MXT-5 column was used as the primary discriminator, while the MXT-1701 column was used as an auxiliary in qualification of compounds. By comparing the calculated retention index of each flavor peak with the ArochemBase database, 27 flavor compounds were identified in the dried gingers processed with different drying methods, and the sensory characteristics of flavor components were obtained in only 100 s. The relative information of the aroma components is presented in Table S2. Of all the flavor components, there were 14 terpenoids, such as zingiberene, *α*-curcumene, *β*-phellandrene, *α*-pinene, *β*-pinene, and myrcene. The relative contents of zingiberene and *β*-phellandrene were highest among ginger aroma, which is consistent with the result by HS-GC-MS. In addition, seven aldehydes, one alcohol, two fatty hydrocarbons, one ester, and two ethers were also identified.

Compared to traditional e-nose, the fast GC e-nose can provide sensory description of each flavor compounds. Generally, spicy and pungency are considered to be the main organoleptic flavors in ginger [31]. As the main contributor in aromatic odor of ginger, zingiberene showed the odor of spicy and pungency through e-nose analysis. Other flavor compounds including myrcene, linalool, terpinen-4-ol, *β*-caryophyllene, *α*-terpinen-7-al, anethole, and propanal, were also smelt as the contributors to spicy scents. Another flavor component with relatively high content was *β*-phellandrene, which was mainly expressed as a minty, fruity, pleasant, herbaceous, and terpenic odor. Sweet, minty, fruity, and citrusy were considered to be the important aromas in ginger or other vegetables and fruits [31,32], and in this study, a total of 21 components exhibited these flavors (Table S2). All the flavors detected in ginger, such as spicy, sweet, minty, citrusy, herbaceous, fruity, and lemon-like., constituted the overall aromatic properties of ginger, and its distinctive odor makes it widely used in spice and functional beverages. Although the types of flavor components in gingers by different drying methods was similar, the abundance of these odors might be different. Table S3 lists the relative contents of flavor components in the samples dried by different drying methods, and Figure 4B shows the changes in odor abundance of different flavor components. Zingiberene and *β*-phellandrene accounting for more than 50% of the total relative content of fragrance components in gingers were important indicators for evaluating the flavor changes of gingers by different drying methods. Compared to FG, the relative content of *β*-phellandrene in the DG was significantly decreased, which indicated that the minty, fruity, pleasant, herbaceous, and terpenic odor of ginger were lost in large quantities after hot air drying. For the relative content of zingiberene, ginger by HAD was significantly higher than those by other drying methods, suggesting that the traditional HAD method allowed the dried gingers to retain more unique spicy and pungent odorants.

#### 3.3.2. Discrimination of the Dried Gingers by PCA and PLS-DA Analysis

The odor fingerprints of ginger by different drying methods were established using the chromatography information of the MXT-5 column and MXT-1701 column (Figure 4A). As shown in the chromatograms, the main flavor peaks were significantly different in FG and DGs by HAD (50, 60, 70 °C), VD (50, 60, 70 °C), SD, and VFD. To further distinguish the differences in gingers by various drying methods, PCA and PLS-DA models were employed.

**Figure 5:**
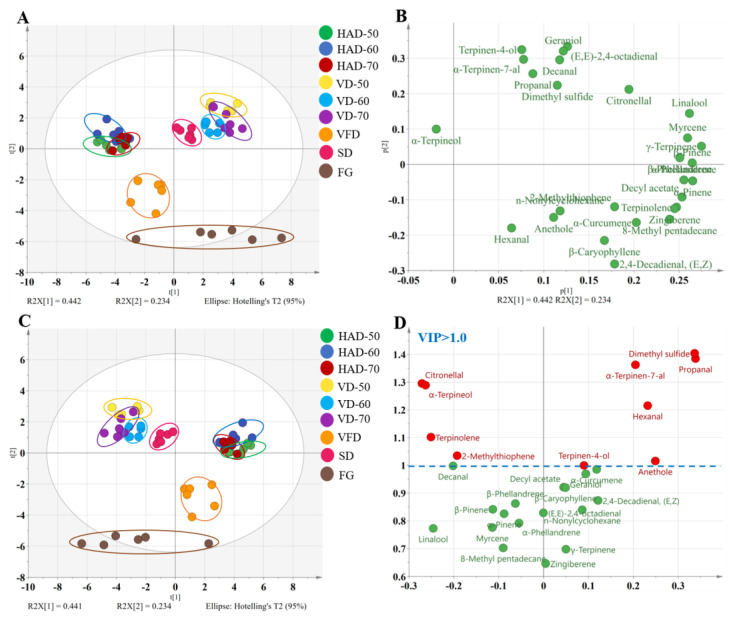
Score plots of the PCA model (**A**) and the variable loading plots (**B**). Score plots of the PLS-DA model (**C**) and V-plots of the variables (**D**) using 27 characteristic sources of odor information obtained by fast GC e-nose in the gingers processed by different drying methods.

The parameters of R^2^X and Q^2^ (cum) in the PCA model were 0.808 and 0.688, respectively, indicating that the model has a strong explanatory and predictive capacity. The separation of FG and DGs dried by HAD, VD, SD, and VFD is clearly displayed in Figure 5A, indicating significant differences in their aroma substances. However, the data points of HAD at 50, 60 and 70 °C, and VD at 50, 60 and 70 °C, partially overlapped, indicating that there was no significant difference in the samples dried at different temperature. The loading scatter plot (Figure 5B) shows that 2,4-decadienal, (E, Z)-, *β*-caryophyllene, *α*-curcumene, and zingiberene contributed greatly to the flavor production of FG, and terpinen-4-ol, *α*-terpinen-7-al, propanal, and geraniol may have had a greater contribution for HAD and SD samples. PLS-DA model was further conducted for all flavor peaks and exhibited a similar distinction to PCA (Figure 5C). The main parameters of R^2^X and Q^2^ (cum) were 0.935 and 0.592, respectively, indicating that the model has a strong explanatory in classification of gingers by different drying methods. To discover the characteristic flavor components in classification, the variables of V-plot (Figure 5D) was profiled. The results showed that the VIP values of 10 flavor components were greater than 1.0, including citronellal, α-terpineol, terpinolene, 2-methylthiophene, terpinen-4-ol, anethole, hexanal, *α*-terpinen-7-al, propanal, and dimethyl sulfide, suggesting that they may be the main aromatic factors in the classification of different drying methods. These VIP components exhibited a spicy, sweet, minty, herbaceous, licorice, moldy, and citrus-like odor and were determined as the most potent odorants in dried gingers.

#### 3.3.3. Flavor Comparison between FG and DG by OPLS-DA

**Figure 6:**
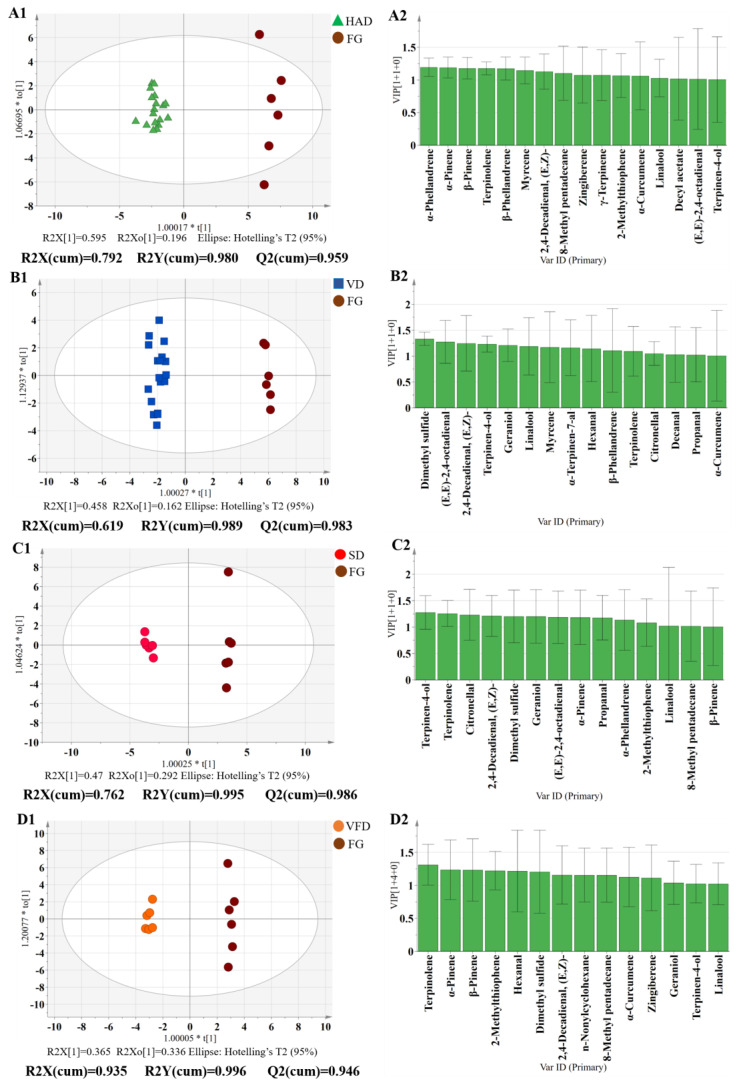
Score scatter plots (**A1**,**B1**,**C1**,**D1**) and VIP plots (**A2**,**B2**,**C2**,**D2**) of the OPLS-DA model using 27 characteristic flavor components of different drying methods for ginger by fast GC e-nose. (**A1**,**A2**): FG vs. DG by HAD; (**B1**,**B2**): FG vs. DG by VD; (**C1**,**C2**): FG vs. DG by SD; (**D1**,**D2**): FG vs. DG by VFD.

PCA and PLS-DA score plots showed that the FG samples were considerably distant from the DG samples, which indicated a completely diverse aroma profile. Therefore, an OPLS-DA model was used to further explain the flavor differentials between fresh and dried gingers, and the scatter plots and flavor variables (VIP > 1.0) are shown in Figure 6. Comparing FG to HAD samples, *α*-phellandrene, *α*-pinene, *β*-pinene, and terpinolene had a greater effect in discrimination, which exhibited the main flavors of sweet, minty, and fruity. The main flavor differentials between FG and VD gingers were dimethyl sulfide, (E, E)-2,4-octadienal decyl acetate, 2,4-decadienal, (E, Z)-, and terpinen-4-ol. Although HAD and VD both had a heating program, the differences in flavor compositions to FG were different. As an advanced drying method, vacuum freeze drying can maintain the nutrients and shape of food products. Ginger by VFD had a significant difference to FG in flavor compounds, such as terpinolene, *α*-pinene, *β*-pinene, and 2-methylthiophene. We also found that only three components were classified as spicy odor among 14 flavor compounds (VIP > 1.0), and this may be attributed to the loss of spicy aroma through VFD. In addition, the traditional sun-dried ginger differed from FG mainly in terms of terpinen-4-ol, terpinolene, citronellal, 2,4-decadienal, and (E, Z)-, and sun-dried ginger exhibited more sweet and fruity scents. Among these variance factors, terpinolene, terpinen-4-ol, 2,4-decadienal, (E, Z)-, and linalool were considered to be the common aroma components between fresh and dried gingers.

### 3.4. Comparative Analysis of HS-GC-MS and Fast GC E-Nose

For comparing the two volatile analysis techniques, dried gingers by four different drying methods (HAD, VD, SD, and VFD) can be significantly distinguished from FG (Figure 3C and Figure 5A), indicating that the flavor composition of FG was greatly altered after the drying process. In terms of four drying methods, gingers by HAD, VD, SD, and VFD were significantly different in aroma profile on the basis of fast GC e-nose, while in HS-GC-MS analysis, ginger by HAD showed similar flavor characteristics to VD samples. The inconsistencies may be attributed to the difference between chromatographic columns and instrument detection efficiency for volatile components. In this study, 52 volatile components were detected on the HP-5MS column in HS-GC-MS, and only 27 were detected on the MXT-column in fast GC e-nose, which demonstrated the various retention of flavor components by different columns; similar results were obtained in the research of Chinese jujube aroma [21]. However, it was notable that 12 major components, namely, *α*-pinene, *β*-pinene, myrcene, *α*-phellandrene, *β*-phellandrene, terpinolene, linalool, citronellal, terpinen-4-ol, *α*-terpineol, *α*-curcumene, and zingiberene were simultaneously identified in ginger by HS-GC-MS and fast GC e-nose, and their chromatographic behaviors were similar. Overall, both HS-GC-MS and fast GC e-nose could achieve the differentiation of ginger by different drying methods using the holistic volatile components. Although more compounds were identified in HS-GC-MS, it could not provide sensory evaluation of these flavor components, while the fast GC e-nose could recognize the main ginger aroma and provide sensory description in a very short time, which made up for the deficiency of GC-MS in flavor evaluation. Therefore, the combination of two techniques can provide a more comprehensive characterization to overall flavor variability of dried gingers by different drying methods.

## 4. Conclusions

In the present study, HS-GC-MS and fast GC e-nose techniques were firstly applied in discriminating gingers by different drying methods based on overall volatile components. A total of 52 volatile components and 27 flavor compounds were identified by HS-GC-MS and fast GC e-nose, respectively. Terpenes were confirmed to be the main volatile constituents of gingers by different drying methods, which accounted for more than 65% and represented the main odor information of ginger. The contents of ginger volatile compounds showed that FG had higher volatile contents than DG, and ginger by HAD retained the richest odor than other drying methods. Due to the effective retention of volatile components, hot air drying is more suitable for the production of DG. Sensory evaluation by fast GC e-nose showed that the ginger flavor was mainly concentrated in the spicy, sweet, minty, fruity, and herbaceous odor. The results of fast GC e-nose analysis also suggested that dried gingers by HAD can retain more unique spicy and pungent odorants, which complemented the HS-GC-MS analysis results. Chemometrics analysis further revealed that fresh ginger and dried gingers by HAD, VD, SD, and VFD were distinguished on the basis of volatile components, indicating that flavor profiles between DG and FG were significantly different. For comparison of fresh and dried gingers, terpinolene, terpinen-4-ol, 2,4-decadienal, (E, Z)-, and linalool were considered as the main variance components. The integration of the two volatile techniques not only provided a comprehensive aroma profile of gingers by different drying methods, but also provided an idea in flavor characterization for drying, storing, and processing of dried ginger or its products.

## Figures and Tables

**Table 1:** Color values of ginger samples by different drying methods.

Parameters	Drying Methods								FG
	HAD-50	HAD-60	HAD-70	VD-50	VD-60	VD-70	VFD	SD	
L*	79.50 ^bc^	79.02 ^c^	76.96 ^e^	78.11 ^d^	79.20 ^c^	77.46 ^de^	86.60 ^a^	80.04 ^b^	77.01 ^e^
a*	0.39 ^e^	1.05 ^c^	1.99 ^a^	1.01 ^cd^	0.60 ^e^	1.50 ^b^	−1.88 ^g^	0.67 ^e^	−1.19 ^f^
b*	32.18 ^de^	32.44 ^de^	33.18 ^bcd^	34.59 ^a^	34.94 ^a^	34.36 ^ab^	33.40 ^bc^	26.18 ^f^	31.42 ^e^
Chroma	32.18 ^ef^	32.46 ^def^	33.24 ^cde^	34.60 ^ab^	34.94 ^a^	34.40 ^abc^	33.46 ^bcd^	26.19 ^g^	31.45 ^f^
Hue angle	89.32 ^c^	88.15 ^ef^	86.58 ^g^	88.33 ^de^	89.01 ^cd^	87.50 ^f^	93.22 ^a^	88.54 ^de^	92.24 ^b^
∆E	3.14 ^e^	3.27 ^e^	3.82 ^d^	4.20 ^d^	4.73 ^c^	4.19 ^d^	9.87 ^a^	6.15 ^b^	------

Different letters (^a–g^) in the same line indicate statistically significant differences (*p* < 0.05, Waller–Duncan’s Text). HAD-50: hot air drying at 50 °C; HAD-60: hot air drying at 60 °C; HAD-70: hot air drying at 70 °C; VD-50: vacuum drying at 50 °C; VD-60: vacuum drying at 60 °C; VD-70: vacuum drying at 70 °C; VFD: vacuum freeze drying; SD: sun drying; FG: fresh ginger. “------” indicates that the ∆E value has not been detected in FG.

**Table 2:** Volatile chemical components identified in gingers by HS-GC-MS.

NO.	RT (min)	Compounds	Type	Formula	CAS	R.Match	RI-m	RI-r
1	4.66	2-Heptanone	ketones	C_7_H_14_O	110-43-0	749	890.3	891
2	4.80	2-Heptanol	alcohols	C_7_H_16_O	543-49-7	859	899.6	901
3	5.24	Tricyclene	monoterpenes	C_10_H_16_	508-32-7	826	922.1	925
4	5.32	*α*-Thujene	monoterpenes	C_10_H_16_	2867-05-2	743	926.1	929
5	5.45	*α*-Pinene	monoterpenes	C_10_H_16_	80-56-8	883	933.0	937
6	5.74	Camphene	monoterpenes	C_10_H_16_	79-92-5	877	947.8	952
7	6.24	Sabinene	monoterpenes	C_10_H_16_	3387-41-5	872	973.0	974
8	6.32	*β*-Pinene	monoterpenes	C_10_H_16_	127-91-3	846	977.3	979
9	6.48	Sulcatone	ketones	C_8_H_14_O	110-93-0	763	985.5	986
10	6.58	*β*-Myrcene	monoterpenes	C_10_H_16_	123-35-3	874	990.6	991
11	6.83	Octanal	aldehydes	C_8_H_16_O	124-13-0	890	1002.6	1003
12	6.91	*α*-Phellandrene	monoterpenes	C_10_H_16_	99-83-2	885	1005.5	1005
13	7.05	3-Carene	monoterpenes	C_10_H_16_	13466-78-9	878	1010.9	1011
14	7.20	*α*-Terpinene	monoterpenes	C_10_H_16_	99-86-5	808	1016.4	1017
15	7.40	p-Cymene	monoterpenes	C_10_H_14_	99-87-6	824	1023.8	1025
16	7.52	*β*-Phellandrene	monoterpenes	C_10_H_16_	555-10-2	864	1028.4	1031
17	7.56	Eucalyptol	alcohols	C_10_H_18_O	470-82-6	873	1030.1	1032
18	7.79	(Z)-Ocimene	monoterpenes	C_10_H_16_	3338-55-4	662	1038.5	1038
19	8.28	*γ*-Terpinene	monoterpenes	C_10_H_16_	99-85-4	824	1057.0	1060
20	9.12	Terpinolene	monoterpenes	C_10_H_16_	586-62-9	882	1088.7	1088
21	9.19	2-Nonanone	ketones	C_9_H_18_O	821-55-6	813	1091.3	1092
22	9.34	α-Naginatene	alkenes	C_10_H_14_O	15186-51-3	703	1097.1	1093
23	9.42	Linalool	alcohols	C_10_H_18_O	78-70-6	788	1100.1	1099
24	9.94	(3E)-4,8-Dimethylnona-1,3,7-triene	alkenes	C_11_H_18_	19945-61-0	755	1115.9	1116
25	10.12	(+)-Sylvestrene	monoterpenes	C_10_H_16_	1461-27-4	790	1121.1	1027
26	10.85	(-)-Camphor	monoterpenes	C_10_H_16_O	464-48-2	840	1143.4	1142
27	10.87	Camphor	monoterpenes	C_10_H_16_O	76-22-2	799	1143.9	1145
28	11.11	Citronellal	aldehydes	C_10_H_18_O	106-23-0	884	1151.1	1153
29	11.55	(-)-Borneol	monoterpenes	C_10_H_18_O	464-45-9	869	1164.6	1166
30	11.95	Terpinen-4-ol	monoterpenes	C_10_H_18_O	562-74-3	697	1176.7	1177
31	12.41	*α*-Terpineol	monoterpenes	C_10_H_18_O	98-55-5	876	1190.7	1189
32	14.18	*β*-Citral	aldehydes	C_10_H_16_O	106-26-3	819	1241.1	1240
33	15.23	*α*-Citral	aldehydes	C_10_H_16_O	141-27-5	885	1270.6	1270
34	15.77	Isobornyl acetate	esters	C_12_H_20_O_2_	125-12-2	793	1285.9	1286
35	16.05	2-Undecanone	ketones	C_11_H_22_O	112-12-9	818	1293.8	1294
36	17.56	*δ*-EIemene	sesquiterpenes	C_15_H_24_	20307-84-0	795	1338.1	1338
37	18.11	Citronellol acetate	esters	C_12_H_22_O_2_	150-84-5	741	1354.2	1354
38	18.56	(+)-Cyclosativene	sesquiterpenes	C_15_H_24_	22469-52-9	805	1367.7	1368
39	18.87	Copaene	sesquiterpenes	C_15_H_24_	3856-25-5	867	1376.6	1376
40	19.40	*β*-Elemene	sesquiterpenes	C_15_H_24_	515-13-9	744	1392.4	1391
41	19.84	Sesquithujene	sesquiterpenes	C_15_H_24_	58319-06-5	879	1405.7	1402
42	20.80	*α*-Bergamotene	sesquiterpenes	C_15_H_24_	17699-05-7	811	1435.8	1435
43	21.47	(E)-*β*-Famesene	sesquiterpenes	C_15_H_24_	18794-84-8	810	1456.9	1457
44	21.59	Alloaromadendren	sesquiterpenes	C_15_H_24_	25246-27-9	834	1460.6	1461
45	22.06	*β*-Chamigrene	sesquiterpenes	C_15_H_24_	18431-82-8	814	1475.4	1476
46	22.30	*α*-Curcumene	sesquiterpenes	C_15_H_22_	644-30-4	864	1483.0	1483
47	22.71	Zingiberene	sesquiterpenes	C_15_H_24_	495-60-3	903	1495.8	1495
48	22.98	*α*-Bulnesene	sesquiterpenes	C_15_H_24_	3691-11-0	769	1506.6	1505
49	23.08	*β*-Bisabololene	sesquiterpenes	C_15_H_24_	495-61-4	816	1511.2	1509
50	23.47	*β*-Sesquiphellandrene	sesquiterpenes	C_15_H_24_	20307-83-9	908	1529.9	1524
51	23.67	(E)-*γ*-Bisabolene	sesquiterpenes	C_15_H_24_	53585-13-0	876	1539.2	1533
52	24.19	Germacrene B	sesquiterpenes	C_15_H_24_	15423-57-1	898	1564.3	1557

RT: retention time; CAS: Chemical Abstracts Service registry number; R.Match: reverse matching scores in NIST library; RI-m: the actual retention index calculated by n-alkanes; RI-r: the theoretical retention index in the NIST 14 library.

## Data Availability

Not applicable.

## Supplementary Materials

The following supporting information can be downloaded at https://www.mdpi.com/article/10.3390/foods11111611/s1, Table S1: Contents of volatile chemical components by different drying methods in gingers by HS-GC-MS. Table S2: The types, formula, and sensory description of flavor components in gingers by fast GC e-nose. Table S3: The relative contents of different drying methods in gingers by fast GC e-nose.
