# Supplementary material for: Comparison of Different Drying Methods on the Volatile Components of Ginger (Zingiber officinale Roscoe) by HS-GC-MS Coupled with Fast GC E-Nose
Source: Foods. 2022 May 30;11(11):1611. doi: 10.3390/foods11111611 (PMC9180836; doi:10.3390/foods11111611)
Supplement: Supplementary file 1 [file foods-11-01611-s001.zip › foods-1735895-supplementary.pdf]

foods

MDPI

Comparison of different drying methods on the volatile components of ginger (*Zingiber officinale* Roscoe) by HS-GC-MS coupled with fast GC e-nose

Dai-Xin Yu [1], Sheng Guo [1,*], Jie-Mei Wang [1], Hui Yan [1], Zhen-Yu Zhang [1], Jian Yang [2] and Jin-Ao Duan [1,*]

[1] National and Local Collaborative Engineering Center of Chinese Medicinal Resources Industrialization and Formulae Innovative Medicine, Jiangsu Collaborative Innovation Center of Chinese Medicinal Resources Industrialization, Nanjing University of Chinese Medicine, Nanjing 210023, China; yudaixin0616@163.com (D.-X.Y.); wjm991106@163.com (J.-M.W.); yanhui@njucm.edu.cn (H.Y.); 15251766992@163.com (Z.-Y.Z.)

[2] State Key Laboratory of Dao-di Herbs Breeding Base, National Resource Center for Chinese Materia Medica, China Academy of Chinese Medical Sciences, Beijing 100700, China; yangchem2012@163.com (J.Y.)

* Correspondence: guosheng@njucm.edu.cn (S.G.); Tel.: +86-25-8581-1916 (S.G.); dja@njucm.edu.cn (J.-A.D.); Tel.: +86-25-8581-1291 (J.-A.D.)

Table S1

Contents of volatile chemical components by different drying methods in gingers by HS-GC-MS

Compounds	Contents (relative to n-decane, μg/g, mean ± SD)								
	HAD-50	HAD-60	HAD-70	VD-50	VD-60	VD-70	VFD	SD	FG
**Terpenes**									
Tricyclene	1.78± 0.4^{b}	1.19± 0.23^{cd}	1.53± 0.15^{bc}	0.75± 0.12^{de}	1.66± 0.29^{b}	1.02± 0.15^{d}	nd	0.39± 0.03^{e}	5.66± 0.68^{a}
α-Thujene	0.22± 0.14^{b}	0.13± 0.03^{b}	0.16± 0.04^{b}	nd	nd	nd	nd	nd	0.65± 0.11^{a}
α-Pinene	43.32± 9.56^{b}	28.08± 5.44^{cd}	37.54± 4.14^{bc}	19.78± 3.25^{de}	39.81± 5.81^{b}	19.49± 10.4^{de}	2.42± 0.58^{f}	10.06± 1.15^{ef}	127.73± 11.63^{a}
Camphene	120.24± 25.96^{b}	83.42± 17.26^{cd}	110.23± 10.56^{bc}	50.38± 9.33^{de}	118.35± 18.6^{bc}	70.69± 13.33^{d}	7.67± 2.16^{g}	33.54± 2.91^{ef}	334.39± 60.28^{a}
Sabinene	3.57± 0.96^{b}	2.27± 0.46^{cd}	2.83± 0.26^{bc}	1.59± 0.36^{de}	3.03± 0.45^{bc}	1.78± 0.45^{d}	nd	0.73± 0.17^{ef}	5.71± 1.18^{a}
β-Pinene	4.5± 1.0^{b}	3.43± 0.67^{b}	4.47± 0.57^{b}	2.08± 0.54^{c}	3.92± 0.88^{b}	2.33± 0.47^{c}	0.19± 0.08^{d}	1.38± 0.17^{c}	10.11± 1.31^{a}
β-Myrcene	21.77± 6.2^{b}	15.87± 2.96^{cd}	20.21± 1.59^{bc}	9.81± 1.92^{ef}	20.73± 3.5^{b}	11.81± 2.2^{de}	1.09± 0.36^{g}	6.67± 0.7^{f}	41.47± 4.67^{a}
α-Phellandrene	23.53± 6.67^{ab}	15.99± 2.51^{de}	18.88± 1.7^{cd}	10.92± 1.92^{f}	21.02± 3.58^{bc}	13.48± 1.7^{ef}	1.32± 0.36^{g}	5.15± 0.35^{g}	27.71± 4.03^{a}
3-Carene	0.69± 0.25^{b}	0.54± 0.14^{bc}	0.67± 0.24^{b}	0.35± 0.14^{cd}	0.7± 0.14^{b}	0.42± 0.12^{cd}	nd	0.21± 0.05^{de}	1.24± 0.15^{a}
α-Terpinene	1.54± 0.52^{a}	1.03± 0.22^{bc}	1.27± 0.2^{ab}	0.56± 0.16^{de}	1.13± 0.16^{b}	0.69± 0.19^{dc}	nd	0.22± 0.06^{ef}	1.59± 0.32^{a}
p-Cymene	1.76±	1.51±	1.89±	0.99±	2±	1.21±	0.18±	0.91±	4.86±

	0.55^{b}	0.33^{bc}	0.13^{b}	0.19^{d}	0.31^{b}	0.49^{cd}	0.05^{e}	0.11^{d}	0.48^{a}
β-Phellandrene	450.82± 150.38^{bc}	311.75± 54.86^{cd}	381.45± 32.1^{bc}	225.55± 33.47^{d}	429.87± 75.29^{bc}	299.64± 59.55^{cd}	31.12± 9.59^{e}	110.54± 9.53^{e}	525.35± 73.16a
(Z)-Ocimene	0.67± 0.45^{b}	0.75± 0.43^{b}	0.57± 0.2^{b}	0.59± 0.27^{b}	1.24± 0.52^{a}	0.49± 0.16^{b}	nd	0.34± 0.12^{bc}	0.77± 0.31^{b}
γ-Terpinene	1.12± 0.23^{b}	0.93± 0.21^{b}	0.99± 0.1^{b}	0.58± 0.14^{c}	1.04± 0.25^{b}	0.6± 0.22^{c}	nd	0.29± 0.09^{d}	1.54± 0.18^{a}
Terpinolene	5.99± 1.86^{b}	5.22± 1.44^{b}	5.93± 0.37^{b}	2.84± 0.74^{cd}	5.42± 0.98^{b}	3.6± 0.73^{c}	0.43± 0.27^{e}	2.14± 0.3^{d}	8.25± 1.01^{a}
(+)-Sylvestrene	0.6± 0.13^{cd}	0.82± 0.09^{bc}	0.77± 0.1^{bc}	0.65± 0.13^{cd}	0.71± 0.27^{bc}	0.64± 0.15^{cd}	0.32± 0.14^{e}	0.48± 0.11^{e}	0.86± 0.08^{a}
(-)-Camphor	nd	nd	nd	nd	nd	nd	nd	nd	1.29± 0.25
Camphor	1.07± 0.3^{ab}	1.11± 0.23^{ab}	0.93± 0.3^{ab}	0.81± 0.12b^{c}	1.12± 0.26^{a}	0.57± 0.21^{c}	0.2± 0.05^{d}	0.56± 0.12^{c}	nd
(-)-Borneol	9.88± 3.35^{ab}	11.38± 1.62^{a}	10.94± 1.74^{a}	8.07± 0.89^{bc}	9.99± 1.33^{ab}	7.76± 1.73^{bc}	5.18± 0.98^{e}	6.33± 0.81^{cd}	8.95± 1.22^{ab}
Terpinen-4-ol	0.76± 0.26^{bc}	0.89± 0.12^{bc}	1± 0.29^{ab}	0.69± 0.14^{cd}	1.2± 0.12^{a}	0.75± 0.24^{cd}	0.31± 0.11^{e}	0.49± 0.29^{e}	0.99± 0.2^{ab}
α-Terpineol	4.3± 1.33^{ab}	4.83± 0.47^{ab}	4.45± 0.98^{ab}	3.53± 1.05^{bc}	4.24± 0.32^{bc}	3.32± 0.98^{cd}	2.08± 0.36^{d}	3.57± 0.65^{bc}	5.29± 1.3^{a}
δ-EIemene	0.66± 0.22^{ab}	0.86± 0.26^{a}	0.8± 0.14^{a}	0.43± 0.14^{c}	0.44± 0.15^{c}	0.4± 0.09^{cd}	nd	0.21± 0.05^{d}	0.52± 0.11^{bc}
(+)-Cyclosativene	3.3± 0.74^{a}	1.5± 1.85^{cd}	1.41± 0.88^{cd}	2.06± 0.6^{bc}	2.73± 0.83^{ab}	2.4± 0.57^{ab}	0.66± 0.29^{d}	1.19± 0.15^{cd}	0.64± 0.27^{d}
Copaene	7.53± 1.18^{ab}	7.46± 1.31^{ab}	7.58± 0.59^{a}	4.76± 0.91^{d}	6.08± 1.57^{cd}	5.42± 1.12^{cd}	1.35± 0.79^{f}	3.02± 0.41^{e}	6.41± 0.6^{bc}
β-Elemene	1.87± 0.83^{bc}	1.96± 0.72^{b}	2.86± 0.72^{a}	0.95± 0.55^{de}	1.2± 0.44^{d}	0.91± 0.6^{de}	0.28± 0.17^{f}	0.6± 0.1^{ef}	1.33± 0.38^{cd}
Sesquithujene	1.46± 0.37^{a}	1.61± 0.38^{a}	1.66± 0.08^{a}	0.83± 0.16^{c}	1.1± 0.27^{b}	0.82± 0.14^{c}	0.21± 0.12^{e}	0.48± 0.09^{d}	1.14± 0.09^{b}
α-Bergamotene	0.75± 0.19^{a}	0.74± 0.19^{a}	0.79± 0.21^{a}	0.4± 0.09^{cd}	0.57± 0.25^{bc}	0.39± 0.15^{d}	nd	0.22± 0.07^{e}	0.66± 0.09^{ab}
(E)-β-Famesene	2.72± 0.54^{a}	2.86± 0.73^{a}	2.25± 0.27^{b}	1.21± 0.37^{c}	1.43± 0.56^{c}	1.02± 0.49^{c}	0.29± 0.1^{d}	0.51± 0.16^{d}	1.98± 0.26^{b}
Alloaromadendren	8.89± 1.75^{a}	8.43± 3.12^{a}	7.58± 0.99^{a}	4.34± 1.16^{b}	5.18± 1.63^{b}	4.28± 1.15^{b}	1.07± 0.67^{c}	2.11± 0.43^{c}	7.41± 0.46^{a}
β-Chamigrene	2.88± 2.05^{b}	5.87± 2.52^{a}	3.16± 1.65^{b}	1.83± 1.75^{bcd}	2.51± 0.67^{bc}	2.02± 1.4^{bcd}	0.4± 0.31^{d}	0.73± 0.41^{cd}	3.52± 2.05^{b}
α-Curcumene	47.49± 10.16^{cd}	58.98± 7.14^{b}	49.13± 2.78^{bcd}	32.91± 12.4^{f}	55.61± 13.97^{bc}	44.09± 14.4^{de}	11.77± 6.74^{g}	35.55± 5.54^{ef}	101.82± 14.82^{a}

Zingiberene	463.07±76.51^{bc}	505.37±117.43^{b}	471.04±25.88^{bc}	314.43±70.16^{d}	392.12±115.97^{cd}	360.83±72.7^{d}	149.84±59.5^{e}	208.78±29.66^{e}	603.56±77.95^{a}
α-Bulnesene	2.19±0.51^{a}	2.12±0.65^{ab}	1.47±0.46^{bc}	1.15±0.42^{cd}	1.51±0.68^{abc}	1.55±0.51^{abc}	0.3±0.13^{e}	0.67±0.29^{de}	2.08±1.25^{ab}
β-Bisabololene	124.15±15.08^{bc}	136.96±25.75^{b}	133.29±7.57^{b}	86.95±22.63^{d}	106.29±32.99^{cd}	89.02±20.07^{d}	37.18±14.23^{e}	57.94±8.25^{e}	181.5±23.61^{a}
β-Sesquiphellandrene	119.93±13.92^{b}	126.09±26.87^{b}	119.96±8.47^{b}	75.75±13.97^{c}	94.02±27.42^{c}	91.57±22.19^{c}	36.79±14.65^{d}	53.97±7.07^{d}	177.41±21.84^{a}
(E)-*γ*-Bisabolene	3.91±0.68^{ab}	3.56±0.7^{b}	3.59±0.7^{b}	2.16±0.23^{c}	2.71±0.98^{c}	2.71±0.58^{c}	0.91±0.57^{d}	1.27±0.36^{d}	4.42±0.61^{a}
Germacrene B	5.47±0.61^{b}	5.93±1.63^{ab}	5.11±0.76^{bc}	3.43±0.55^{d}	4.21±1.29^{cd}	3.64±0.79^{d}	1.34±0.63^{e}	2.07±0.41^{e}	6.82±1.14^{a}
**Ketones**									
2-Heptanone	0.35±0.08^{b}	0.3±0.04^{b}	0.28±0.05^{b}	0.33±0.07^{b}	0.29±0.08^{b}	0.24±0.11^{b}	nd	0.26±0.07^{b}	3.29±0.48^{a}
Sulcatone	0.41±0.18^{b}	0.56±0.11^{b}	0.99±0.09^{b}	0.85±0.11^{b}	0.49±0.17^{b}	0.3±0.12^{b}	nd	1.35±0.2^{b}	30.34±7.14^{a}
2-Nonanone	1.66±0.7^{b}	1.71±0.39^{b}	1.4±0.36^{bc}	0.99±0.43^{cd}	1.2±0.28^{cd}	0.88±0.38^{d}	nd	0.43±0.19^{e}	4.34±0.43^{a}
2-Undecanone	2.71±1.1^{b}	2.94±0.61^{b}	2.25±0.89^{bc}	1.58±0.75^{c}	1.96±0.37^{c}	1.67±0.54^{c}	0.31±0.12^{d}	0.41±0.24^{d}	3.85±0.84^{a}
**Aldehydes**									
Octanal	0.81±0.36^{a}	0.68±0.16^{ab}	0.63±0.26^{abc}	0.57±0.27^{abc}	0.55±0.36^{bcd}	0.36±0.18^{d}	nd	0.38±0.04^{cd}	nd
Citronellal	2.32±0.52^{a}	1.98±0.27^{a}	1.34±0.18^{b}	1.35±0.55^{b}	0.97±0.18^{c}	1.15±0.35^{bc}	0.27±0.12^{de}	0.62±0.35^{d}	nd
β-Citral	22.02±4.08^{b}	28.07±3.76^{a}	27.2±2.19^{a}	11.57±1.65^{d}	19.17±4.29^{bc}	18.11±3.08^{c}	8.38±2.21^{de}	6.78±0.65^{e}	19.23±6.86^{bc}
α-Citral	37.21±9.88^{b}	44.55±7.13^{a}	33.74±3.63^{b}	11.1±2.22^{e}	18.39±3.03^{d}	25.21±9.25^{c}	11.31±2.69^{e}	6.53±0.63^{e}	33.39±10.04^{b}
**Alcohols**									
2-Heptanol	1.91±0.45^{de}	2.11±0.34^{d}	1.87±0.19^{de}	3.14±0.57^{b}	2.56±0.31^{c}	2.11±0.55^{d}	0.43±0.15^{f}	1.51±0.24^{e}	7.12±0.51^{a}
Eucalyptol	76.04±16.26^{b}	76.21±11.81^{b}	85.36±8.89^{b}	50.85±7.89^{c}	79.9±11.4^{b}	51.18±6.12^{c}	16.92±5.43^{d}	57.08±7.4^{c}	188.87±36.32^{a}
Linalool	4.27±1.59^{cd}	4.62±1.14^{bc}	5.51±0.96^{b}	4.79±0.76^{bc}	4.92±1.37^{bc}	3.26±0.55^{d}	1.64±0.68^{e}	3.54±0.51^{d}	10.91±0.85^{a}
**Esters**									
Isobornyl acetate	2.06±0.21^{a}	2.24±0.35^{a}	2.23±0.18^{a}	1.16±0.43^{cd}	2.16±0.41^{a}	1.53±0.22^{b}	0.89±0.3^{d}	1.15±0.15^{cd}	1.4±0.34^{bc}
Citronellol acetate	0.43±0.17^{a}	0.43±0.22^{a}	0.4±0.11^{a}	0.19±0.06^{b}	0.18±0.07^{b}	0.25±0.16^{b}	nd	nd	nd

**Alkenes**									
α-Naginatene	0.93±	0.9±	1.25±	1.18±	0.84±	0.44±	0.26±	0.99±	0.63±
	0.48^{abc}	0.37^{bcd}	0.21^{a}	0.2^{ab}	0.4^{cd}	0.24^{ef}	0.13^{f}	0.3^{abc}	0.12^{de}
(3E)-4,8-dimeth ylnona-	0.52±	0.41±	0.57±	0.44±	0.52±	0.36±	0.16±	0.25±	0.64±
1,3,7-triene	0.3^{ab}	0.22^{abc}	0.33^{a}	0.19^{abc}	0.22^{ab}	0.18^{abc}	0.06^{c}	0.09^{bc}	0.26^{a}

[1] Notes: nd, not detected. Different letter (a–g) in the same line indicate statistically significant differences ($p < 0.05$, Waller-Duncan's Text). Data are represented as the mean ± SD (n=6). HAD-50: hot air drying at 50 °C; HAD-60: hot air drying at 60 °C; HAD-70: hot air drying at 70 °C; VD-50: vacuum drying at 50 °C; VD-60: vacuum drying at 60 °C; VD-70: vacuum drying at 70 °C; VFD: vacuum freeze drying; SD: sun drying; FG: fresh ginger.

Table S2

The types, formula, and sensory description of flavor components in gingers by fast GC e-nose.

NO.	Compounds	Formula	CAS	RI-m, MXT-5	RI-r, MXT-5	RI-m, MXT-1701	RI-r, MXT-1701	Sensory Description
1	Propanal	C_3H_6O	123-38-6	486	499	570	579	Spicy; Nutty; Plastic; Cocoa
2	Dimethyl sulfide	C_2H_6S	75-18-3	508	509	590	573	Sweet; Cabbage; Gasoline; Onion; fruity
3	2-Methylthiophene	C_5H_6S	554-14-3	778	775	798	827	Sweet; Onion; Green; Gasoline
4	Hexanal	$C_6H_{12}O$	66-25-1	803	801	894	890	Sweet; Fresh; Fruity; Green; Herbaceous
5	α-Pinene	$C_{10}H_{16}$	80-56-8	951	937	942	945	Sweet; Camphor; Citrus; Fruity; Green
6	β-Pinene	$C_{10}H_{16}$	127-91-3	970	979	967	994	Sweet; Pine; Resinous; woody
7	Myrcene	$C_{10}H_{16}$	123-35-3	995	996	1012	1025	Spicy; Nutty; Plastic; Balsamic
8	α-Phellandrene	$C_{10}H_{16}$	99-83-2	1021	1004	1029	1029	Spicy; Minty; Citrus; Green
9	β-Phellandrene	$C_{10}H_{16}$	555-10-2	1049	1031	1063	1059	Minty; Herbaceous; Pleasant; Fruity; Terpenic
10	γ-Terpinene	$C_{10}H_{16}$	99-85-4	1073	1060	1111	1089	Sweet; Citrus; Fruity; Gasoline; Herbaceous; Lemon
11	Terpinolene	$C_{10}H_{16}$	586-62-9	1073	1088	1111	1112	Fruity; Green; Juicy; anisic
12	Linalool	$C_{10}H_{18}O$	78-70-6	1105	1107	1199	1198	Spicy; Anise; Citrus; Floral; Fragrant
13	(E, E)-2,4-Octadienal	$C_8H_{12}O$	30361-28-5	1119	1115	1253	1247	Fruity; Cucumber; Melon; Seaweed
14	Citronellal	$C_{10}H_{18}O$	106-23-0	1161	1158	1263	1259	Sweet; Aldehydic; Citrus; Lemon
15	Terpinen-4-ol	$C_{10}H_{18}O$	562-74-3	1171	1177	1282	1272	Spicy; Pepper; Herbaceous; Licorice; Moldy
16	α-Terpineol	$C_{10}H_{18}O$	98-55-5	1196	1189	1300	1300	Minty; Anise; Lilac; Citrus; Floral
17	Decanal	$C_{10}H_{20}O$	112-31-2	1214	1219	1310	1315	Sweet; Lemon; Orange; Aldehydic; Burnt; Citrus
18	Geraniol	$C_{10}H_{18}O$	106-24-1	1257	1255	1378	1376	Sweet; Citrus; Rose; Floral; Fruity
19	α-Terpinen-7-al	$C_{10}H_{14}O$	1197-15-5	1284	1282	1408	1415	Spicy; Fatty
20	2,4-Decadienal, (E,Z)-	$C_{10}H_{16}O$	25152-83-4	1300	1295	1434	1432	Deep-fried; Fatty; Fried; Geranium; Green

21	Anethole	$C_{10}H_{12}O$	4180-23-8	1311	1310	1442	1459	Spicy; Anise; Herbaceous; Licorice; Sweet
22	Decyl acetate	$C_{12}H_{24}O_2$	112-17-4	1412	1408	1487	1479	Fruity; Citrus; Fresh; Orange; Soapy
23	*β*-Caryophyllene	$C_{17}H_{28}$	87-44-5	1486	1482	1501	1514	Spicy; Fruity; Green; Musty; Sweet
24	*α*-Curcumene	$C_{15}H_{22}$	644-30-4	1505	1483	---	---	Fruity; Herbaceous
25	Zingiberene	$C_{15}H_{24}$	495-60-3	1517	1495	---	---	Spicy; Pungent
26	8-Methyl pentadecane	$C_{16}H_{34}$	22306-28-1	1545	1545	54.09	1542	---
27	n-Nonylcyclohexane	$C_{15}H_{30}$	2883-2-5	1549	1556	1565	1570	---

[1] Note: RI-m, MXT-5 and RI-m, MXT-1701: Retention index measured by n-alkanes in column MXT-5 or MXT-1701; RI-r, MXT-5 and RI-r, MXT-1701: the reference of theoretical retention index in two different columns. CAS: Chemical Abstracts Service registry number.

Table S3

The relative contents of different drying methods in gingers by fast GC e-nose.

Compounds	relative contents (%, mean ± SD)								
	HAD-50	HAD-60	HAD-70	VD-50	VD-60	VD-70	VFD	SD	FG
β-Phellandrene	25.23±0.01^{e}	29.77±0.02^{d}	31.00±0.01^{d}	42.11±0.01^{b}	45.10±0.00^{a}	44.8±0.00^{a}	45.73±0.00^{a}	41.31±0.01^{b}	36.59±0.00^{c}
Zingiberene	26.24±0.23^{a}	21.63±0.07^{b}	22.60±0.03^{b}	14.38±0.04^{cd}	12.66±0.02^{de}	12.28±0.04^{e}	15.50±0.12^{c}	15.75±0.02^{c}	16.34±0.03^{c}
β-Pinene	3.58±0.03^{g}	6.50±0.04^{f}	6.33±0.02^{f}	9.52±0.02^{d}	10.70±0.01^{bc}	12.44±0.00^{a}	7.52±0.02^{e}	9.83±0.01^{cd}	10.92±0.02^{b}
n-Nonylcyclohexane	9.30±0.03^{a}	7.97±0.03^{ab}	6.79±0.02^{bc}	5.31±0.01^{cde}	4.53±0.01^{de}	3.58±0.01^{e}	5.55±0.01^{cde}	4.63±0.02^{cde}	6.19±0.02^{bcd}
Myrcene	2.18±0.14^{f}	2.97±0.31^{e}	3.03±0.25^{e}	4.31±0.30^{ab}	4.24±0.18^{b}	4.52±0.20^{a}	3.52±0.46^{c}	4.15±0.21^{b}	3.29±0.23^{d}
α-Phellandrene	2.12±0.28^{d}	2.22±0.99^{d}	2.34±0.80^{d}	3.36±0.71^{b}	3.77±0.43^{a}	3.64±0.47^{a}	3.87±0.78^{a}	3.04±0.61^{c}	3.63±0.40^{ab}
α-Pinene	1.16±0.07^{f}	1.89±0.20^{e}	1.77±0.20^{e}	3.15±0.10^{c}	3.75±0.08^{b}	4.15±0.11^{a}	2.63±0.24^{d}	2.91±0.12^{cd}	4.29±0.16^{a}
8-Methyl pentadecane	4.32±0.07^{a}	3.64±0.23^{b}	3.80±0.15^{b}	2.44±0.07^{d}	2.07±0.09^{e}	2.02±0.09^{e}	2.56±0.43^{cd}	2.63±0.10^{cd}	2.85±0.15^{c}
α-Terpinen-7-al	6.35±0.76^{a}	6.01±2.12^{a}	5.16±1.69^{b}	3.05±0.18^{cd}	2.98±0.66^{cde}	2.80±0.99^{cde}	2.69±3.86^{de}	3.25±0.99^{c}	2.49±1.57^{e}
α-Curcumene	3.77±0.01^{ab}	3.19±0.05^{ab}	3.44±0.04^{ab}	1.99±0.00^{ab}	1.75±0.00^{b}	1.62±0.02^{b}	2.12±0.06^{ab}	2.38±0.02^{ab}	5.09±0.06^{a}
Geraniol	3.39±0.08^{a}	3.06±0.08^{b}	2.67±0.06^{c}	1.95±0.02^{d}	1.53±0.01^{e}	1.47±0.03^{e}	1.28±0.07^{e}	2.03±0.05^{d}	1.31±0.06^{e}
Linalool	2.03±0.13^{a}	2.15±0.19^{a}	2.05±0.10^{a}	1.82±0.06^{b}	1.50±0.02^{c}	1.49±0.07^{c}	1.40±0.11^{c}	1.80±0.08^{b}	1.39±0.08^{c}

α-Terpineol	2.47±0.02^{b}	2.67±0.08^{ab}	3.02±0.06^{a}	1.27±0.01^{c}	1.03±0.01^{c}	1.07±0.03^{c}	1.35±0.06^{c}	1.45±0.03^{c}	1.07±0.05^{c}
Propanal	0.09±0.14^{a}	0.07±0.06^{b}	0.06±0.02^{bc}	0.06±0.02^{bc}	0.04±0.01^{d}	0.03±0.02^{e}	0.03±0.05^{e}	0.05±0.04^{cd}	0.03±0.05^{e}
Dimethyl sulfide	0.98±0.04^{a}	0.37±0.17^{c}	0.17±0.08^{d}	0.70±0.02^{b}	0.61±0.08^{b}	0.45±0.05^{c}	0.33±0.06^{c}	0.15±0.08^{d}	0.06±0.05^{d}
2-Methylthiophene	0.16±0.22^{a}	0.15±0.71^{ab}	0.15±0.22^{ab}	0.11±0.05^{ce}	0.08±0.03^{e}	0.07±0.08^{e}	0.08±0.31^{e}	0.11±0.13^{ce}	0.13±0.67^{bc}
Hexanal	0.11±0.04^{a}	0.08±0.14^{b}	0.08±0.17^{b}	0.05±0.02^{c}	0.04±0.02^{c}	0.04±0.03^{c}	0.04±0.07^{c}	0.09±0.05^{b}	0.09±0.07^{b}
γ-Terpinene	0.37±0.13^{a}	0.36±0.38^{ab}	0.35±0.18^{ab}	0.33±0.09^{ab}	0.34±0.06^{ab}	0.33±0.13^{abc}	0.28±0.21^{c}	0.33±0.20^{abc}	0.31±0.17^{bc}
Terpinolene	0.72±0.24^{ab}	0.65±0.59^{b}	0.56±0.31^{c}	0.67±0.11^{b}	0.50±0.12^{cd}	0.45±0.17^{d}	0.50±0.69^{cd}	0.51±0.29^{cd}	0.78±0.35^{a}
(E,E)-2,4-octadienal	0.52±0.07^{a}	0.51±0.03^{a}	0.49±0.04^{a}	0.34±0.01^{bc}	0.29±0.02b^{cd}	0.28±0.02^{cd}	0.25±0.07^{d}	0.35±0.03^{b}	0.18±0.01^{e}
Citronellal	0.49±0.12^{a}	0.45±0.12^{a}	0.44±0.08^{a}	0.45±0.03^{a}	0.27±0.04^{b}	0.32±0.05^{b}	0.31±0.07^{b}	0.46±0.07^{a}	0.24±0.24^{b}
Terpinen-4-ol	1.06±0.12^{a}	0.92±0.07^{b}	0.98±0.07^{ab}	0.65±0.04^{c}	0.47±0.04^{d}	0.49±0.04^{d}	0.29±0.05^{e}	0.74±0.01^{c}	0.32±0.20^{e}
Decanal	0.90±0.08^{a}	0.87±0.06^{a}	0.81±0.06^{a}	0.53±0.05^{b}	0.41±0.02^{c}	0.40±0.03^{c}	0.40±0.08^{c}	0.52±0.05^{b}	0.37±0.15^{c}
2,4-Decadienal, (E,Z)-	0.59±0.07^{a}	0.44±0.22^{b}	0.36±0.22^{cd}	0.31±0.10^{d}	0.32±0.09^{d}	0.28±0.08^{d}	0.42±0.42^{bc}	0.31±0.21^{d}	0.60±6.19^{a}
Anethole	0.51±0.36^{a}	0.38±0.70^{ab}	0.37±1.38^{ab}	0.21±0.50^{cd}	0.20±0.64^{cd}	0.18±0.76^{d}	0.23±2.57^{bc}	0.24±1.33^{bc}	0.36±2.25^{abc}
Decyl acetate	0.88±0.07^{a}	0.74±0.14^{ab}	0.82±0.25^{ab}	0.66±0.09^{ed}	0.60±0.10^{ed}	0.58±0.12^{e}	0.78±0.43^{ab}	0.73±0.23^{bc}	0.70±0.41^{bc}
β-Caryophyllene	0.46±0.18^{a}	0.34±0.30^{bc}	0.35±3.33^{abc}	0.26±0.20^{cd}	0.22±0.21^{d}	0.20±1.75^{d}	0.33±0.90^{bc}	0.25±2.25^{cd}	0.38±0.88^{ab}

[1] Different letter (a–g) in the same line indicate statistically significant differences ($p < 0.05$, Waller-Duncan's Text). Data are represented as the mean ± SD (n=6). HAD-50: hot air drying at 50 °C; HAD-60: hot air drying at 60 °C; HAD-70: hot air drying at 70 °C; VD-50: vacuum drying at 50 °C; VD-60: vacuum drying at 60 °C; VD-70: vacuum drying at 70 °C; VFD: vacuum freeze drying; SD: sun drying; FG: fresh ginger.
